# The fungal elicitor eutypine from *Eutypa lata* activates basal immunity through its phenolic side chains

**DOI:** 10.1093/hr/uhac120

**Published:** 2022-06-01

**Authors:** Pingyin Guan, Florian Schmidt, Jochen Fischer, Michael Riemann, Eckhard Thines, Peter Nick

**Affiliations:** College of Horticulture, China Agricultural University, Beijing 100193, China; Molecular Cell Biology, Botanical Institute, Karlsruhe Institute of Technology, Fritz-Haber-Weg 4, 76131 Karlsruhe, Germany; Institut für Biotechnologie und Wirkstoff-Forschung gGmbH, Hanns-Dieter-Hüsch-Weg 17, 55128 Mainz, Germany; Institut für Biotechnologie und Wirkstoff-Forschung gGmbH, Hanns-Dieter-Hüsch-Weg 17, 55128 Mainz, Germany; Molecular Cell Biology, Botanical Institute, Karlsruhe Institute of Technology, Fritz-Haber-Weg 4, 76131 Karlsruhe, Germany; Institut für Biotechnologie und Wirkstoff-Forschung gGmbH, Hanns-Dieter-Hüsch-Weg 17, 55128 Mainz, Germany; Molecular Cell Biology, Botanical Institute, Karlsruhe Institute of Technology, Fritz-Haber-Weg 4, 76131 Karlsruhe, Germany

## Abstract

Grapevine trunk diseases (GTDs) affect grape production and reduce vineyard longevity worldwide. Since the causative fungi also occur in asymptomatic trunks, we address disease outbreak in terms of altered chemical communication between host and endophyte. Here, we identified four chemically similar secondary metabolites secreted by the GTD-associated fungus *Eutypa lata* to analyse their modes of action in a grapevine cell culture of *Vitis rupestris*, where microtubules were tagged by GFP. Treatment with the metabolite eutypine activated defence responses, evident from extracellular alkalinisation and induction of defence genes. Eutypinol, instead, eliminated microtubules, in contrast to the other three compounds. Furthermore, we evaluated the effect of four corresponding chemical analogues of these compounds, sharing the phenolic but lacking the alkyne moiety. These analogues were able to induce similar defence responses in *V. rupestris* cells, albeit at reduced amplitude. Since closely related moieties differing only in details of the side groups at the phenolic ring differ significantly with respect to the response of the host cell, we propose that these fungal compounds act through a specific binding site at the membrane of grapevine cells. We corroborate this specificity by combination experiments, where the eutypine and the eutypinol analogues behave competitively with respect to the elicited responses. In summary, *Eutypa lata* secretes compounds that elicit host defence in a specific manner by interfering with early events of immunity signalling. This supports the notion that a real understanding of GTDs has to address inter-organismic chemical communication.

## Introduction

Over the past decade, Grapevine Trunk Diseases (GTDs) have become a major destructive threat to modern viticulture worldwide. These diseases result in severe economic losses due to decreased cumulative yield and shortened profitable longevity of the vines [1], as well as increased management costs and reduced wine quality [2]. In contrast to most plant diseases, GTDs do not follow Koch’s postulates. Notably, GTDs do not meet the first postulate, which states that the parasitic organism must be found in all cases where the disease is observed, and that the disease does not develop where the parasitic organism is absent. A classical approach in plant protection is to kill the potential pathogen with toxic chemicals. In the case of GTDs, this was achieved in the past using arsenite, but this practice has been banned in Europe because of its toxicity to humans [3]. This plant protection strategy has a negative ecological footprint (as it is also toxic to non-human species). Moreover, intoxication of a pathogen that does not conform with Koch’s postulates does not represent a meaningful strategy. However, the occurrence of GTDs is increasing in vineyards worldwide as consequence of climate change. Since conventional approaches fail to control this type of disease [4], there is an urgent need for new strategies to suppress the outbreak of symptoms.

One of the major forms of GTDs is *Eutypa* Dieback, associated with the fungus *Eutypa lata*, which infects and colonises the xylem tissue from fresh pruning wounds and then spreads to the cambium and phloem of grapevine trunks [5, 6]. Specific symptoms include dwarf and withered shoots, necrosis of leaf margins, and wilting inflorescences. The entire plant may die following several years of infection. Neither the annual canes nor the leaves of infected plants contain any mycelia. Thus, the fungus in the infected trunk seems to emit phytotoxic compounds to the distal parts of the plant. These compounds might be secondary metabolites [7] or cell wall-degrading enzymes [8, 9]. In addition to phytotoxic compounds, phytopathogenic fungi can secrete so-called effectors, i.e. small molecules that suppress the defence of the host [10]. Since the host usually exhibits a defence response, one must assume that fungus associated elicitors play a role as well. Generally, upon contact with GTD associated fungi, the infected plant responds by biosynthesis of antifungal proteins and phenolic phytoalexins accumulating in the xylem [11, 12]. The timing and amplitude of these defence responses decides about the outcome of the interaction, as has been shown recently during a comparative study on a further type of GTD, Botryosphaeriaceae related Dieback [13].

In case of *E. lata*, the main secreted secondary metabolites are acetylenic phenols and heterocyclic analogues [14]. Some have been isolated and characterised from culture filtrates of *E. lata*. For instance, eutypine [15, 16] was first considered as the principal phytotoxin responsible for foliar symptoms during infection [17]. Further analysis of culture filtrates identified additional secreted products, including eutypinol, *O*-methyleutypine, *O*-methyleutypinol (a eutypine analogue with a carboxylic side chain), siccayne, eulatinol, and eulatachromene as well as their derivatives [7, 14, 18]. Culture filtrates from *E. lata* can activate basal immunity in grapevine cell cultures. Principally, such filtrates may contain fungal cell wall remnants, secondary metabolites and proteins. However, since autoclaved culture filtrates (where proteins should be denatured) induce similar immunity responses as unprocessed filtrates, the activation of plant defence seems to be rather elicited by small molecules, such as secondary metabolites [19]. In fact, activity-guided fractionation, screening for the ability to elicit defence responses in grapevine cells, such as extracellular alkalinisation and the transcription of phytoalexin-synthesis genes identified the polyketide O-methylmellein as amplifier of grapevine defence [19, 20].

Most secondary metabolites reported for *E. lata* have similar chemical structures deriving from eutypine. For instance, eutypinol (where the methanone side group of eutypine is reduced to the alcoholic form), or siccayne (where the methanone side group is replaced by a hydroxyl group) were found in the secretome of an *E. lata* strain that activated significant defence responses [20]. The functional context of these compounds is not clear, however.

To identify compounds that trigger or modulate defence responses, activity-guided fractionation has been a powerful strategy. For instance, fractionation of microbial culture extracts by ion-exchange chromatography allowed to identify pathogen-associated molecular patterns (PAMPs) from bacteria, fungi and oomycetes [20]. In our previous work, we followed a similar approach in a particular strain of *Eutypa lata*. Upon cultivation in different media, the culture filtrates differed in their ability to activate defence responses in a grapevine cell system [20]. Fractionation through preparative HPLC led to a fraction able to evoke a strong defence response. This fraction harboured O-methylmellein, a compound that by itself did not elicit defence, but was able to strongly amplify a defence response triggered by the bacterial elicitor flg22. During the same fractionation, we discovered an additional peak in the neighbourhood that was able to induce transcripts of phenylammonium lyase, the first committed step of the phenylpropanoid pathway that gives rise to both, lignin and the major phytoalexins in grapevine, the stilbenes. In this peak, eulatinol and siccayne were abundant, leading to the question, whether these compounds might act as elicitors. We have dissected the signal transduction deployed by bacterial elicitors for grapevine cells in detail [20, 21]: In basal immunity, a rapid influx of calcium precedes apoplastic oxidative burst triggered by the membrane bound NADPH oxidase Respiratory burst oxidase Homologue. These primary events activate mitogen-activated protein kinase (MAPK) cascades, culminating in the upregulation of transcripts for phytoalexin-synthesis genes, and accumulation of the stilbene glycoside α-piceid concomitant with the activation of jasmonate biosynthesis and signalling [22]. For cell-death related immunity, calcium influx and oxidative burst represent early events as well. However, they occur in the reversed temporal order (burst is first, calcium influx is later). While MAPK cascades and induction of phytoalexin transcripts are observed in a similar manner as for basal immunity, the resulting stilbenes accumulate mainly as aglycons (α-piceid) and oxidised oligomers (viniferins) [23]. In addition, a rapid remodeling of actin filaments in the cortical cytoplasm heralds subsequent programmed cell death involving specific metacaspases [20, 24]. This detailed map of early defence responses allows to characterise fungal compounds with respect to their functional targets in grapevine immunity.

**Figure 1:**
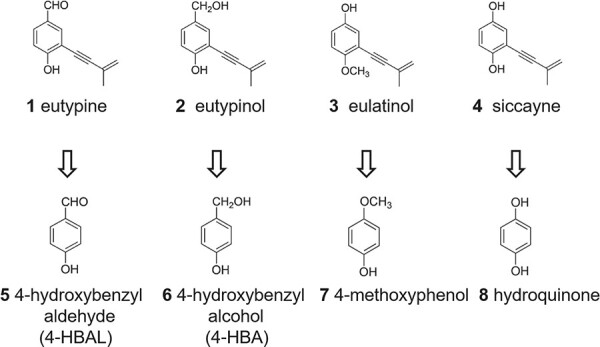
Structures of *Eutypa lata* fungi produced metabolites and their analogues

In the current study, we aimed to analyse the potential mode of action for structurally similar secondary metabolites from *E. lata* with respect to plant defence. We first examined the response to the *E. lata* acetylenic phenols eutypine, eutypinol, siccayne and eulatinol in a grapevine line, where microtubules were observable due to a GFP tag. We then mapped the cellular events elicited by chemical analogues of these secondary metabolites (differing just in the presence/absence of the alkyne moiety; Fig. 1) to assign the bioactivity to either the acetylenic or the phenolic moieties. We can show that eutypine can elicit defence responses, and that this depends on the phenolic moiety, but not on the acetylic side chain. The eutypine analogue 4-HBA also induced defence response in grapevine and tomato leaves. We further show that the aldehyde group at the phenolic ring is responsible for the activation of defence. Upon reduction to an alcoholic group, the activity is strongly diminished. This specific pattern, along with results from competition experiments suggests that eutypine is not acting as a toxin, but as a signal.

## Results

### Eutypine specifically elicits extracellular alkalinisation

**Figure 2:**
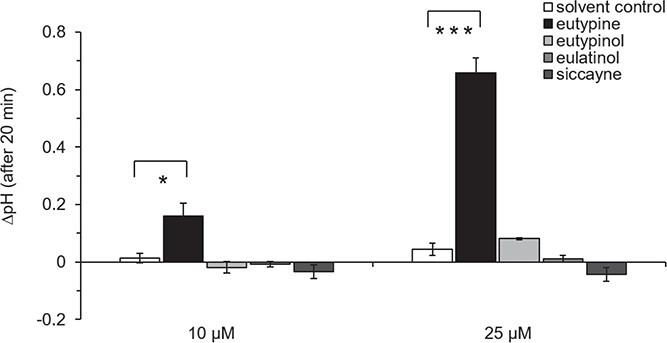
Extracellular alkalinisation of grapevine model *V. rupestris* TuB6-GFP to acetylenic phenols secreted by *Eutypa lata.* Treatments of two concentrations (10 μM and 25 μM) of eutypine, eutypinol, eulatinol, and siccayne were conducted for one hour. 0.1% and 0.25% methanol were respectively used as the corresponding solvent control. The extracellular pH changes (∆pH) were recorded via a pH meter. Data represent mean ∆pH (after 20 min treatments) ± and standard error (SE) from at least five independent experimental series, each in technical triplicates. Significant differences are indicated by ^*^ (*P* < 0.05), ^**^ (*P* < 0.01), or ^***^ (*P* < 0.001) based on a homoscedastic Student’s t-test.

To evaluate the effects of *E. lata* secondary metabolites on grapevine defence, we monitored calcium influx as one of the earliest defence responses [23]. We measured extracellular alkalinisation as a readout for calcium influx [25] in response to secreted compounds from *E. lata* in cultivated cells from *V. rupestris* TuB6-GFP. Compared with the solvent control, eutypine induced a clear, quick pH response, reaching a maximum of 0.16 (at 10 μM) and 0.66 pH units (at 25 μM) after around 20 min (Fig. 2). In contrast, none of the other three tested metabolites, eutypinol, eulatinol and siccayne, was able to induce any significant pH changes at any of these concentrations although their chemical structures are very similar to eutypine (Fig. 1). Along with the dose-dependence observed for eutypine, this indicates that the response to eutypine is specific. We, therefore, decided to investigate downstream cellular events in response to eutypine.

### Eutypine specifically induces the expression of defence genes

**Figure 3:**
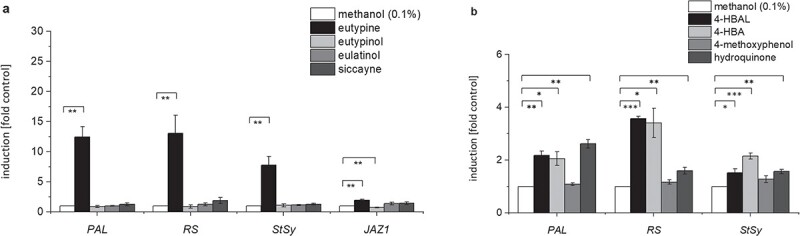
Response of defence genes in the cellular grapevine model *V. rupestris* TuB6-GFP to acetylenic phenols secreted by *Eutypa lata.* Eutypine, eutypinol, eulatinol, and siccayne were administered in a concentration of 10 μM for one hour. 4-HBAL, 4-HBA, 4-methoxyphenol, and hydroquinone were administered in 50 μM for one hour. Treatment with 0.1% methanol was used as solvent control. The induction of defence genes (*PAL*, *RS*, *StSy*, *JAZ1*) was measured by qPCR. Data represent mean ± standard error (SE) from independent experimental series, each in technical triplicates. Significant differences are indicated by ^*^ (*P* < 0.05), ^**^ (*P* < 0.01), ^***^ (*P* < 0.001), based on a homoscedastic Student’s t-test.

The perception of elicitors generally leads to the induction of defence-related genes. In our grapevine cell system, transcripts for the phytoalexin-synthesis genes *PAL*, *RS*, *StSy*, and the jasmonate-response gene *JAZ1* are induced swiftly [23]. We, therefore, measured the transcript levels of these genes in *V. rupestris* cell cultures treated with *E. lata* metabolites by RT-qPCR, using cultures treated with solvent only (0.1% methanol) as controls. Eutypine treatment induced significant expression of *PAL* and *RS*, up to 12-fold (Fig. 3a). Eutypine also raised *StSy* transcript levels 8-fold over control levels (Fig. 3a). By contrast, eutypinol failed to induce the transcription of those defence genes and even suppressed the transcription of *JAZ1* by about 40% relative to controls (Fig. 3a). Likewise, eulatinol and siccayne treatment hardly modulated gene expression. For instance, siccayne caused up-regulation of *RS* transcription by 1.8-fold relative to solvent controls, but this increase in transcript levels did not reach statistical significance (Fig. 3a). Thus, the mode of action of structurally similar metabolites on the expression of basal-immunity-related genes differed clearly. These results indicate that the different substituent groups of the phenolic ring of eutypine and its derivatives may play a role in activating grapevine basal immune responses.

### Eutypine, eutypinol and siccayne cause only minor cell mortality

**Figure 4:**
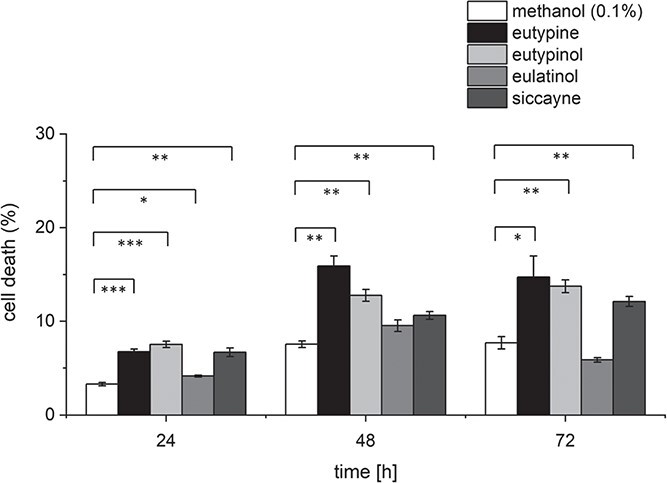
Response of mortality in the cellular grapevine model *V. rupestris* TuB6-GFP to acetylenic phenols secreted by *Eutypa lata.* Eutypine, eutypinol, eulatinol, and siccayne were administered in a concentration of 10 μM for the indicated time intervals. Treatment with 0.1% methanol was used as solvent control. Mortality was scored using the Evans Blue dye exclusion assay. Data represent means ± SE from 1500 individual cells sampled in three independent experimental series. Significant differences are indicated by ^*^ (*P* < 0.05), ^**^ (*P* < 0.01), or ^***^ (*P* < 0.001) based on a homoscedastic Student’s t-test.

Eutypine was reported to be one of the critical toxins that cause the grapevine foliar symptoms induced by infection by GTDs, based on its apparent toxic effect on leaves [17]. However, a later study determined that eutypine was not the main product of pathogenic isolates of *E. lata* [26]. Instead, it was eutypinol that was detected in almost all pathogenic *E. lata* isolates at high levels, although it did not act as a toxin [26, 27]. Likewise, siccayne and eulatinol were reported to be tolerated by grapevine leaves [28]. To get insight into a potential phytotoxicity of the secreted fungal metabolites, we followed cell mortality by the Evans Blue Dye Exclusion Assay in response to 10 μM of each metabolite. Eutypine caused a minor, but significant cell mortality after 24 h, reaching around 7% dead cells, compared to 3% for cells treated with the solvent (0.1% methanol) only (Fig. 4). Cell death had reached a plateau of more than 15% from 48 h of treatment (Fig. 4). In contrast to the literature report, also eutypinol and siccayne induced a significant, albeit lower cell mortality. For example, cell cultures treated with siccayne or with eutypinol for 48 h displayed 11% and 13% dead cells, respectively (Fig. 4). By contrast, 10 μM of eulatinol showed little toxicity to grapevine cells. The cell death in response to eutypine, eutypinol and siccayne developed slowly, requiring more than a day to manifest. This speaks against an acute phytotoxicity of these compounds.

### Eutypinol specifically eliminates cortical microtubules

**Figure 5:**
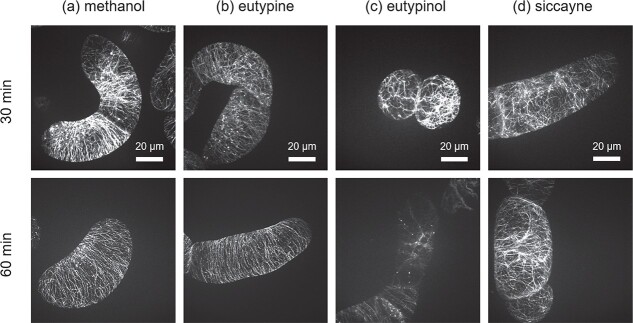
Response of microtubules in the cellular grapevine model *V. rupestris* TuB6-GFP to acetylenic phenols secreted by *Eutypa lata.* Eutypine (b), eutypinol (c), and siccayne (d) were administered in a concentration of 10 μM for the indicated time intervals. Treatment with 0.1% methanol (a) was used as solvent control. For each treatment, a representative confocal section from a z-stack along with two time points, visualization of GFP fused with microtubule, cortical microtubules were shown. Observations are representative of at least four independent experimental series with a population of 50 individual cells for each treatment. Bars, 20 μm.

A re-organisation of the cortical microtubules belongs to the early responses to elicitors, especially in the context of effector triggered immunity [29]. In our previous work we found that culture extracts from *E. lata* induced significant microtubule de-polymerisation, but the responsible compound was not identified [20]. To test a potential effect on microtubules, we exposed *V. rupestris* cells expressing the fluorescent tubulin marker GFP-*At*TUB6 with 10 μM of either eutypine, eutypinol or siccayne for 30 min and 60 min, or with 0.1% methanol as the solvent control, and visualised the microtubule network by confocal spinning-disc microscopy. While in the solvent control numerous cortical microtubules were aligned perpendicular to the long cell axis (Fig. 5a), eutypinol caused microtubules to disappear. This elimination had already initiated at 30 min after addition of eutypinol. At 60 min, almost all cortical microtubules had disappeared (Fig. 5c). In contrast to the activity with respect to gene expression (Fig. 3a), eutypine left microtubules intact (Fig. 5b). Likewise, siccayne did not affect the microtubule network (Fig. 5d). Thus, eutypine, which was the compound that activated defence genes most efficiently, did not eliminate microtubules, while eutypinol, which was able to eliminate microtubules, was ineffective in the activation of defence genes.

Although eutypinol caused significant microtubule elimination within one hour (Fig. 5c), it did not cause relevant mortality (Fig. 4), as it should result, if such an important structure as microtubules is absent over a longer time interval. We, therefore, assessed the microtubules after a prolonged treatment for 8 h. To our surprise, the microtubule network had returned to full restoration after 8 h (Fig. S1). Thus, the elimination was transient and followed by establishment of a new network that seemed to be resilient against the effect of eutypinol.

### Analogues for eutypine and eutypinol activate calcium influx

**Figure 6:**
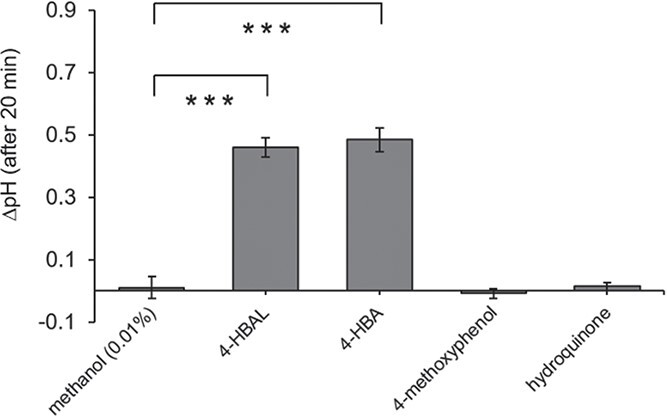
Extracellular alkalinisation of *V. rupestris* TuB6-GFP to chemical analogues of *Eutypa lata* secreted metabolites. Cells were incubated with 50 μM 4-hydroxybenzyl aldehyde (4-HBAL), 4-hydroxybenzyl alcohol (4-HBA), 4-methoxyphenol or hydroquinone for an hour. 0.01% methanol was used as the corresponding solvent control. The extracellular pH changes (∆pH) were recorded via a pH meter. Data represent mean ∆pH (after 20 min treatments) ± and standard error (SE) from at least five independent experimental series. Significant differences are indicated by ^*^ (*P* < 0.05), ^**^ (*P* < 0.01), or ^***^ (*P* < 0.001) based on a homoscedastic Student’s t-test.

To identify the functional moiety that acts as a ligand in the acetylenic phenolics secreted by *E. lata*, we measured the response to treatments with chemical analogues differing in the presence/absence of the alkyne moiety of eutypine (4-hydroxybenzyl aldehyde or 4-HBAL), eutypinol (4-hydroxybenzyl alcohol or 4-HBA), eulatinol (4-methoxyphenol) and siccayne (hydroquinone) (Fig. 1). As rapid readout for defence, we measured apoplastic alkalinisation reporting calcium influx [25]. While the solvent control (0.01% methanol), was not able to elicit any change of pH (Fig. 6), we observed a strong response of around 0.5 units after treatment with the eutypine analogue 4-HBAL and the eutypinol analogue 4-HBA. By contrast, the eulatinol analogue 4-methoxyphenol, and the siccayne analogue hydroquinone were not able to deploy extracellular alkalinisation. The solvent control, treatment time and cell line of Fig. 6 were same as Fig. 2. Comparing with the responses to their acetylated counterparts (Fig. 2), the efficacy of 4-HBAL is roughly half that of eutypine, since 25 μM of eutypine elicited a pH shift of 0.6 units, while 50 μM of 4-HBAL yielded around 0.5 units. Interestingly, 4-HBA seems to be more potent than its natural template – 25 μM of eutypinol yielded a response of less than 0.1 pH units (Fig. 2), while 50 μM of 4-HBAL produced almost 0.5 pH units (Fig. 6). Thus, the effect of the alkyne moiety on the biological effect seems to be antagonistic in these compounds – stimulating bioactivity in case of eutypine, restraining bioactivity in case of eutypinol.

**Figure 7:**
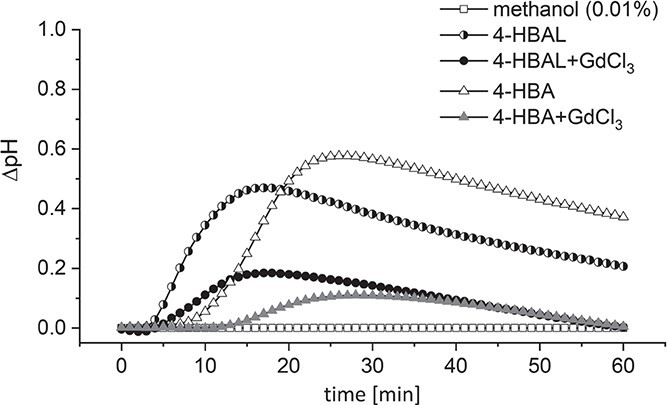
Effect of GdCl_3_ on apoplastic alkalinisation induced by 4-HBAL and 4-HBA in *V. rupestris* TuB6-GFP. Extracellular pH was evaluated in response to 50 μM 4-HBAL, 4-HBA alone or separately combined with 100 μM GdCl_3_. 0.01% methanol as solvent control. The extracellular pH changes (∆pH) were recorded via a pH meter. Data represent mean ∆pH from at least five independent experimental series.

To test, whether the extracellular alkalinisation in response to 4-HBAL and 4-HBA reported calcium influx, we treated *V. rupestris* cell cultures with 100 μM of the calcium-channel blocker GdCl_3_ together with 50 μM 4-HBAL or 4-HBA and monitored pH changes. We observed that GdCl_3_ inhibited extracellular pH responses to both, 4-HBAL and 4-HBA: GdCl_3_ lowered the peak in pH change induced by 4-HBAL by 62% from 0.47 to 0.18 at 18 min, and the pH response activated by 4-HBA by 81% from 0.58 to 0.11 in 26 min (Fig. 7). Thus, both compounds seem to deploy calcium influx. We noticed that the response to 4-HBA initiated later (lag time < 10 min, peak 25 min) as compared to the response to 4-HBAL (lag time < 5 min, peak 15 min).

### Analogues for eutypine and eutypinol activate phytoalexin transcripts

**Figure 8:**
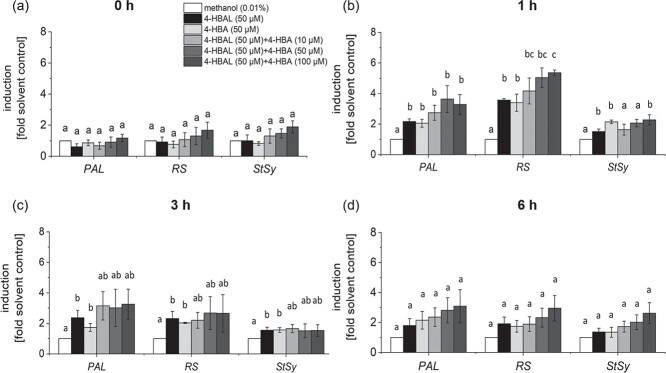
Response of defence genes in the cellular grapevine model *V. rupestris* TuB6-GFP to chemical analogues of acetylenic phenols secreted by *Eutypa lata.* Cells were treated with 50 μM 4-HBAL, 4-HBA or 4-HBAL crossed with three concentrations of 4-HBA: 10 μM, 50 μM, 100 μM, for 0 to 6 hours with sequential equivalent-time sampling (a, b, c, d). The 0.01% methanol was used as the solvent control. The transcription of defence genes (*PAL*, *RS*, *StSy*) was recruited via qPCR. Data represent mean ± standard error (SE) from three independent experimental series, each in technical triplicates. Different lowercase letters indicate the significance at P = 5% (Student’s t-test).

Because both, 4-HBAL and 4-HBA, activated calcium influx, an early signal in plant defence, we wondered, whether they can also activate defence transcripts. To test their mode of interaction, we also tested different combination of the two compounds, whereby a constant concentration of 4-HBAL (50 μM) was complemented by 10 μM, 50 μM or 100 μM of 4-HBA. We followed steady-state transcript levels for *PAL*, *RS* and *StSy* by RT-qPCR over time (0, 1, 3, and 6 h). In fact, both, 4-HBAL and 4-HBA treatment caused a mild, but significant up-regulation of the three genes (Fig. 3b). The largest amplitude (around 3.5-fold of the resting level) occurred for *RS*, while *PAL* (around 2-fold), and *StSy* (around 1.5–2-fold) were less responsive. The solvent control, treatment time and cell line of Fig. 3b were same as Fig. 3a. Compared to the response to eutypine (Fig. 3a), the analogue 4-HBAL induced around 20–25% of the response. Interestingly, 4-HBA was of comparable efficiency, although its template, eutypinol was not effective at all (compare Fig. 3b and Fig. 3a). Interestingly, also hydroquinone, the analogue of siccayne, induced a significant response of PAL, which was as strong as that of 4-HBAL and 4-HBA (Fig. 3b). The effect of 4-HBAL and 4-HBA remained transient, easing off gradually (Fig. 8c, d). At 6 h after addition of the compound, the transcript levels, although still higher than in the control, had stopped to be significantly different (Fig. 8c, d). When we combined the two analogues, we saw a dose-dependent increase of transcript levels. However, only for *RS* became this enhancement over the single compounds significant. Even under this condition (Fig. 8b), the resulting induction (around 5.5-fold) was less than half (around 12-fold) seen for the template eutypine (Fig. 3a). The two analogues did not interact additively. The addition from 4-HBA to the level seen for 4-HBAL was clearly lower than if one would sum up the responses of the individual compounds. This was not due to a saturation of the system, since the levels achieved by the alkylinated template eutypine were much higher, demonstrating that the induction was still far from its maximal amplitude. The alkyne moiety seems to have a different effect, though. In one case, the alkyne moiety seems to enhance the effect of the phenolic moiety, since 4-HBAL was less effective than eutypine. In the second case, the alkyne moiety seems to quell the effect of the phenolic moiety, since 4-HBA was effective, while eutypinol was not.

To test, whether the induction of phytoalexin synthesis transcripts by 4-HBAL and 4-HBA might be the consequence of a phytotoxic effect, we followed cell mortality in response to either 50 μM of 4-HBAL, 50 μM of 4-HBA, or the combination thereof, using 0.01% methanol as the solvent only control. However, none of these treatments resulted in any significant cell mortality if scored after 24 h (Suppl. Fig. S2). We did observe a slight increase (about 18%) in cell death when cells were treated with 50 μM 4-HBAL after 48 h. However, this did not reach statistical significance. Therefore, we conclude that the induction of phytoalexin-synthesis transcripts by these analogues of eutypine and eutypinol is not due to a phytotoxic effect.

Moreover, we have checked whether the eutypine analogue 4-HBAL can activate similar defence response in the grapevine (*Vitis vinifera* L.Tianyuanqi) and tomato (*Lycopersicon esculentum* cv. Micro-Tom) leaves. In agreement with the above findings, the one-hour treatment of 500 μM 4-HBAL on grapevine leaves have induced significant expression of genes *PAL* and *StSy*, indicating that the basal immunity is partly evoked (Suppl. Fig. S3). However, the dose–response curve showed that the concentrations effective in cell culture, are not sufficient for leaf discs (Suppl. Fig. S3). In addition, 4-HBAL also elicited a strong expression of defence gene *LeSOD* (up to 12-fold) in tomato (Suppl. Fig. S4). In summary, our data have clearly indicated that 4-HBAL is able to elicit grapevine defence response and tomato immunity.

## Discussion

This work was motivated by the concept that Grapevine Trunk Diseases (GTDs) differ from classical diseases because they are conditional, depending on environmental conditions, such as drought stress (Botryosphaeria Dieback: Galarneau et al., 2019; Esca Syndrome: Lima et al., 2017) [30, 31]. This would explain, why the progression of these diseases correlates with Global Climate Change. To understand and, eventually, contain, diseases of this type, it is important to dissect the chemical interaction between host and fungus. These signals are most likely secondary metabolites produced by fungi, and probably arise in response to other chemical signals, either from competing or cooperating fungi or from the host itself. If the host is shifted under stress (such as drought), this chemical interaction is perturbed, which culminates in the formation of the toxins causing so called apoplectic breakdown including the characteristic foliar symptoms that constitute a terminal manifestation of GTDs [32]. In our previous work, we had used bioactivity-guided fractionation to identify a compound released by a specific strain of *Eutypa lata*, when shifted from a Potato Dextrose (PDA) to a Biotin-Aneurin-Folic Acid (BAF) medium. This compound, O-methylmellein, was not able to deploy plant defence by itself, but it amplified defence elicited by the bacterial elicitor flg22 [20]. In the current study, we analysed the mode of action for four additional compounds that had been isolated from the same screen. Although these secondary compounds (eutypine, and its derivatives eutypinol, eulatinol, and siccayne) were structurally closely related, only one of them, eutypine, was able to activate an immune response. Using chemical analogues sharing the phenol, but lacking the alkyne moieties, we could show that the activation of defence was linked with the presence of an aldehyde residue at a side group of the phenolic ring. If this aldehyde residue was reduced to an alcohol, the activity was lost. Interestingly, this difference was not observed in the analogues, where the alkyne moiety was missing. On the other hand, only eutypinol was able to trigger a (reversible) elimination of microtubules. Thus, closely related moieties differing only in details of the side groups at the phenolic ring differ significantly with respect to the response of the host cell, and this difference depends on presence or absence of the alkyne moiety. These findings stimulate the following questions: What is the function of these secreted compounds – are they toxins, or are they modulators of plant immunity? Are these compounds acting as signals, hijacking the regulation of a host function for the sake of the fungus? How can the secretion of compounds modulating defence of the host be integrated into the biology of the fungus?

### Eutypine elicits grapevine immunity

Eutypine has been originally identified during an activity-guided fractionation for phytotoxic activity using grapevine protoplasts as experimental model [17]. However, the claim of phytotoxicity is based on concentrations that were significantly higher (around 80 μM) than those used here. In concentrations comparable to those of the current study (10 μM), the mortality was minor, consistent with our data. There have been claims that eutypine causes thylakoid dilations and chloroplast swellings [33], but this was based on treatments with 750 μM of eutypine, i.e. concentrations that are almost two orders of magnitude higher than those of the current study. Likewise, the frequently purported claim that eutypine is taken up by a non-saturated ion-trapping mechanism [34] is based on experiments, where uptake of radioactively labelled eutypine into grapevine cells was measured upon incubation with eutypine in the mM range measuring an uptake rate in the range of nM per minute, casting doubt on the specificity of the assay (moreover, since the cells had not been washed before going to the scintillation counter). The same holds true for the claim that eutypine targets mitochondria because the reported changes of oxidation rates seen for eutypine exceeding 100 μM were minor (around 20%), despite the mitochondria were isolated. The same concerns hold true for the claim that eutypine is suppressing anthocyanin synthesis in a grapevine cell line [35], because eutypine concentrations had to be raised above 200 μM to see this effect. Comparative studies, where different strains of *E. lata* were compared with respect to their metabolites [26, 28] showed that, while eutypinol was consistently produced, eutypine was only detected in some of the extracts. While this is casting further doubt on a role of eutypine as phytotoxin, fairness commands to mention that eutypinol, which seems to lack any phytotoxic effect, is the main breakdown product, when eutypine is fed to grapevine cells [36], which would be consistent with a scenario, where the plant host tries to de-toxify a pathogen virulence factor. However, this would hold true for any virulence factor and does not mean that this factor qualifies as phytotoxin. In fact, alternative compounds such as eulatachromene or 2-isopropenyl-5-formyl benzofuran [37] have been proposed as true agents for phytotoxicity. As summary, the claims that eutypine acts as phytotoxin must be seen with a certain scepticism. The current study as well does not lend any evidence whatsoever for a function of eutypine as phytotoxin.

If eutypine is not a phytotoxin, it might act as a signal. In fact, this is supported by the activation of extracellular alkalinisation (Fig. 2), a proxy for calcium influx [25]. A similar alkalinisation in response to eutypine has also been reported for sugar beet leaf discs and pulvini of *Mimosa pudica* [38]. Eutypine can, in addition, activate a weak but significant defence-related transcripts (Fig. 3a). A molecule or an event can become a signal, if it conveys information that is meaningful in the appropriate context. In other words, signals are signals, because they are specific. We find that the cellular events elicited by eutypine are highly specific, because they are exclusively induced by eutypine, but not by the structurally very related eutypinol, nor by eulatinol, nor by siccayne.

Specificity is also seen in the response of the host – eutypine is inducing the transcripts for the phytoalexin-synthesis genes, while the jasmonate-response gene *JAZ1* remains mostly silent (Fig. 3a), contrasting with the response by the bacterial PAMP flg22, which can activate both, phytoalexin synthesis genes and *JAZ1* [22]. This indicates that eutypine can deploy a part of the signalling deployed by flg22, but not the full set of responses. The flg22 has induced significant *PAL* gene expression at a concentration of 1 μM [20]. For eutypine, when the concentration increased to 10 μM, the induction level of *PAL* gene is weak but significant. The most straightforward explanation is therefore to interpret eutypine as a weak elicitor that deploys a part of basal immunity.

### Eutypine might interfere with a membrane-located receptor

Elicitors do not have characteristic structure. Elicitors include molecules released from or produced by pathogens that guide plants to perceive and recognize signal from pathogens by receptors, leading to the initiation of plant immunity [39]. Elicitors in pathogens have been divided into two categories: general elicitors and race-specific elicitors [40]. General elicitors are molecules that participate in normal defence signaling pathways, while race-specific elicitors are avirulence factors related to R gene-mediated signaling [41, 42]. The operational definition of an elicitor is based on two criteria. First, it requires a specific binding site; second, it induces signalling culminating in defence reactions. Typically, general elicitors interact with specific binding sites located at the membrane, such as FLS2, the receptor for the bacterial elicitor flg22 [43], or chitin oligosaccharide elicitor- binding protein (CEBiP), the receptor for fungal chitin [44]. This leads to early signalling involving calcium influx, oxidative burst, and activation of MAP kinase signalling that conveys the signal to the nucleus, where transcriptional activators deploy the expression of phytoalexin synthesis genes, but also other pathogenesis related transcripts. That eutypine can activate defence signalling and defence responses, has been already discussed above. Now, we will discuss the first criterion, namely, whether eutypine exhibits specificity of binding. There are several arguments in favour of such a specificity:

The response to eutypine depends on small chemical details of the phenolic moiety. Eutypine can deploy extracellular alkalinisation as proxy for calcium influx, eutypinol, eulatinol, and siccayne cannot (Fig. 2). This is exactly matched by the pattern seen for the phytoalexin synthesis transcripts (Fig. 3a). The activity depends on the presence of a methyl-aldehyde side group of the phenolic ring (Fig. 1), if it is reduced to an alcohol (eutypinol), or replaced by a hydroxyl residue (eulatinol, siccayne), the activity is gone. In contrast to calcium influx and activation of defence transcripts, microtubules are eliminated exclusively eliminated by eutypinol (Fig. 5), not by eutypine, nor by siccayne, which adds a further level of specificity.

The specificity depends on the presence of the alkyne moiety as to be concluded from the comparison of the natural compounds with their analogues harbouring only the phenolic moiety of these compounds (Fig. 1). While only eutypine, but not eutypinol can activate extracellular alkalinisation (Fig. 2), 4-hydroxybenzyl alcohol (the analogue of eutypinol) is as efficient as 4-hydroxybenzyl aldehyde (the analogue of eutypine) in doing so (Fig. 6). Thus, the above-mentioned impact of the methyl-aldehyde side group requires the presence of the alkyne moiety. Again, this is reflected in the pattern of defence transcripts (Fig. 3b), where both analogues activate to the same extent. For PAL, even the siccayne analogue hydroquinone becomes active (although it is not activating calcium influx, indicating a divergent signalling chain), while the eulatinol analogue 4-methoxyphenol is not active. This indicates that the presence of a terminal oxygen at the phenolic side group enables binding *per se*, which is suppressed by the alkyne moiety, in case of a hydroxylic group (eutypinol, siccayne), but permitted in case of an aldehyde group (eutypine).

Since both, 4-hydroxybenzyl alcohol and 4-hydroxybenzyl aldehyde can activate phytoalexin synthesis transcripts, they should interact additively, if they were sensed by different binding sites. However, when we did a combination experiment (Fig. 8b), we observed that the effects of 4-hydroxybenzyl alcohol and 4-hydroxybenzyl aldehyde remained far below the level expected for such an additive interaction, indicating that they compete for the same binding site, whose abundance is limited. This binding site is likely to be found at the surface of the plasma membrane since membrane permeation of these compounds is very low. Experiments with radioactively labelled eutypine [34] report uptake in the range of nM after incubation of mM, i.e. concentrations that are six orders of magnitude higher. Perforation of the membrane is not very likely either, because the mortality induced by the natural compounds (Fig. 4) as well as of their chemical analogues (Fig. 8) is hardly detectable.

The molecular nature of this binding site is rewarding to be elucidated, because it will shed light on the functional context of eutypine and the host process that is hijacked by this compound. A recent study has identified a quinon receptor that would meet several operational criteria of the eutypine binding site. This Cannot Respond to DMBQ1 (CARD1) receptor deploys calcium influx, is localised in the membrane, activates the chitin signalling culminating in induction of plant defence genes, and is triggered by fungal quinone compounds that are produced by fungi to support laccases, i.e. the enzymes that help to mobilise lignin as carbon source [45]. This leucine-rich repeat receptor like kinase also exists in grapevine (UniProt F6HJY5, gene locus 12s0035g02090) and binds the structurally related lignin breakdown product acetosyringone.

While eutypinol is not active with respect to calcium influx and activation of defence genes, it is active with respect to microtubule elimination. It should be noted that eutypinol was induced in *E. lata* upon cultivation in BAF medium, correlating with an activity of the culture filtrate against microtubules [20]. Reorganisation of microtubules in response to fungal infection is a well-known phenomenon [46]. Whether the eutypine/eutypinol binding site can trigger two concurrent signalling pathways, one involving calcium influx and activation of phytoalexin synthesis transcripts, the other leading to microtubule elimination, represents a rewarding topic for future research. On the other hand, the effect of the combined eutypine and entypinol on microtubule network might also be an interesting topic.

## Conclusion and outlook

This work provides links between the chemical structure and the biological activity of fungal secondary metabolites. We investigated the mode of action of four secondary metabolites (eutypine, eutypinol, eulatinol and siccayne) isolated from *E. lata*, a fungus associated with GTDs. Although these metabolites have highly similar structures, they show significant differences with respect to their ability to elicit defence responses in our grapevine cell system. These differences depend on small chemical details at the phenolic group and on the presence of the alkyne moiety, indicative of a specific binding site on the surface of the plant cells.

Genome sequencing of fungal organisms has shown that GTD-related fungi harbour extensive gene clusters for secondary metabolism, although they secrete only few compounds in axenic cultures [47]. The list of secondary metabolites produced by *E. lata* given in Table 1 may therefore represent only the “tip of the iceberg”. While the recent progress in fungal genomics harbour enormous potential for the discovery of new secondary metabolites, we need to elucidate the (host-derived?) signals able to awaken these sleeping metabolic potencies.

**Table 1:** Secondary metabolites produced by fungi *Eutypa lata*

	class of compound	structure
eutypine [15, 16, 28]	phenols	1; Figure 1
eutypinol [16, 28]		2; Figure 1
eulatinol [16, 28]		3; Figure 1
siccayne [16, 28]		4; Figure 1
O-methyleutypine [16]		9; Figure S5
O-methyleutypinol [16]		10; Figure S5
eutypin carboxylic acid analogue [16]		11; Figure S5
3-(3,4-dihydroxy-3-methyl-1-butynyl)-4-hydroxy-benzaldehyde [16]		12; Figure S5
2-(3,4-dihydroxy-3-methyl-1-butynyl)-4-hydroxymethyl-phenol [16]		13; Figure S5
3-(3,4-dihydroxy-3-methyl-1-butynyl)-4-hydroxy-benzoic acid [16]		14; Figure S5
epoxyexahydrochromanones [48]		15a; Figure S5
epoxyexahydrochromanones [48]		15b; Figure S5
eulatachromene [28]		16; Figure S5
eutypoxide B [49]	cyclohexene epoxide	17; Figure S5
allenicepoxycyclohexane [16]		18; Figure S5
2-isopropenyl-5-formylbenzofuran [16, 26]	miscellanea	19; Figure S5
O-methylmellein [20]	isocoumarins	20; Figure S5

## Materials and methods

### Cell line and plant materials

We used a suspension cell line of *Vitis rupestris* expressing the *Arabidopsis thaliana* β-tubulin TuB6 fused with the green fluorescent protein (GFP) at the N-terminus, under control of the Cauliflower Mosaic Virus (CaMV) 35S promoter [45]. Suspension cells of this line, termed Vrup TuB6-GFP, were grown in full-strength Murashige and Skoog (MS) liquid medium (Duchefa Biochemie, The Netherlands) supplemented with 30 g/L sucrose, 200 mg/L KH_2_PO_4_, 100 mg/L *myo*-inositol, 1 mg/L thiamine, and 0.2 mg/L 2,4-D, pH 5.8. We sub-cultured the cells weekly by complementing 6 mL of stationary cells up to 30 mL fresh medium in 100 mL Erlenmeyer flasks. The cultures remained at 26°C under constant shaking (150 rpm) on a KS260 basic orbital shaker (IKA Labortechnik, http://www.ika.de). We maintained selective stringency by adding 30 mg/L hygromycin to the medium. If not stated otherwise, data represent three independent experimental series with cells collected at the peak of the proliferation phase (at day 4 after sub-culture).

The *V. vinifera* L. Tianyuanqi grown in the greenhouse of Shang Zhuang Experimental Station, China Agricultural University, Beijing. The Micro-Tom tomato (*L. esculentum*) seeds were purchased from the PanAmerican Seed Company, Chicago, IL, USA.

### Fungal metabolites and chemical analogues

We obtained the *E. lata* secondary metabolites eutypine, eutypinol, siccayne and eulatinol by fermentation of *Eutypa lata*, strain IBWF E16121 in 20 liters of BAF medium as described in Guan *et al.* (2020) [20]. We dissolved the fungal metabolites in 100% methanol to a stock of 10 mM, diluting to a working concentration of 10 μM, if not specified otherwise. The chemical homologues of the fungal metabolites, 4-HBAL (4-hydroxybenzyl aldehyde), 4-HBA (4-hydroxybenzyl alcohol), 4-methoxyphenol and hydroquinone were all purchased from Sigma-Aldrich (Merck, Darmstadt, Germany) and prepared in 2% methanol to stocks of 10 mM stocks and used at a final concentration of 50 μM, if not stated otherwise.

### Apoplastic Alkalinisation

Calcium influx can be evaluated by measuring apoplastic alkalinisation as a readout [25]. To measure the dose–response of calcium influx induced by fungal metabolites, we inoculated cells with eutypine, eutypinol, eulatinol or siccayne at a concentration of either 10 μM or 25 μM. We added methanol as the solvent to a final concentration of 0.1% or 0.25% to control cells. To determine the effect of the analogues of the fungal metabolites on cellular calcium influx, we treated the cells with 50 μM of either 4-HBAL, 4-HBA, 4-methoxyphenol, or hydroquinone, along with a solvent control (0.01% methanol). To verify, whether the observed alkalinisation induced by the chemical analogues 4-HBAL- or 4-HBA was due to proton co-import in the context of calcium influx we used 100 μM of the calcium-channel inhibitor GdCl_3_ added together with the analogue. Again, 0.01% methanol served as solvent control.

The resulting apoplastic alkalinisation was examined via a pH meter (Schott handy lab, pH 12) equipped with a pH electrode (Mettler Toledo, LoT 403-M8-S7/120). A paperless readout (VR06; MF-Instruments GmbH, Albstadt-Truchtelfingen, Germany) recorded pH over time. We quantified the difference between treatment and mock controls using the peak values. We pre-equilibrated the cells for at least 1 h on an orbital shaker, before treatment with various compounds. Data represent mean and standard errors from at least five independent experimental series.

### RNA extraction and RT-qPCR

We measured steady-state transcript levels for different defence-related genes after treating 10 mL of cell suspension for 1 h with 10 μM of either eutypine, eutypinol, eulatinol, or siccayne. In a different set of experiments, we administered the chemical analogues, 4-HABL or 4-HAB, both at 50 μM for 1 h. In a third set of experiments, 50 μM of 4-HABL were supplemented with increasing concentrations (10 μM, 50 μM, 100 μM) of 4-HBA for different time intervals between 0 and 6 h. For the plant leave experiments, 50 μM, 500 μM or 5000 μM of 4-HABL were used to treat grapevine and tomato leaves. In all experiments, solvent controls, consisting of 0.01% methanol, were included.

We extracted total RNA from the *V. rupestris* TuB6-GFP cells, grapevine leaves or tomato leaves using the Universal RNA Purification Kit (Roboklon, Germany) following the instructions of the manufacturer. Subsequently, we digested the RNA on column with DNase I (Qiagen, Hilden, Germany), before checking RNA quality by electrophoresis on a 0.8% agarose gel. We determined RNA concentration spectrophotometrically (NanoDrop, Radnor, USA) at wavelengths of 230, 260 and 280 nm. First-strand cDNA synthesis was initiated from 1 μg total RNA using the M-MuLV cDNA Synthesis Kit (New England Biolabs; Frankfurt am Main, Germany) based on the protocol of the manufacturer.

We performed quantitative RT-PCR (RT-qPCR) on a CFX96TM real-time PCR cycler (Bio-Rad, USA) for three genes of the grapevine phenylpropane phytoalexin pathway, namely, phenylalanine ammonia lyase (PAL), stilbene synthase (StSy), and resveratrol synthase (RS). In addition, the jasmonate response factor ZIM/tify-domain protein 1 (JAZ1) was included as readout for the status of grapevine basal immunity. Relative transcript accumulation for tomato genes of superoxide dismutase (SOD), β-1,3-glucanase (PR2b; E) and chitinase (CHI9), isochorismate synthase (ICS), the non-inducible pathogenesis-related 1 (NPR1), TGA transcription factors 1a (TGA1a), TGA transcription factors 2 (TGA2), pathogenesis-related protein gene 1b1 (PR1b1), and pathogenesis-related protein gene 5 (PR5) were also measured. The values for these genes were normalised to the relative transcript levels of the housekeeping genes *elongation factor 1α* (*EF1*α) or *LeActin* as internal standards, respectively. **Supplementary Table S1** gives the details of primer sequence and PCR conditions. The 2^-ΔΔCt^ method served to calculate relative expression levels. Each data point represents three independent experimental series, each in technical triplicates.

### Live-cell imaging

We employed spinning-disc microscopy to image microtubule responses to various compounds from *E. lata*. We treated Vrup TuB6-GFP cells with 10 μM of either eutypine, eutypinol, or siccayne for 30 min or 60 min, respectively. The microtubule network was visualised with an AxioObserver Z1 microscope (Zeiss, Jena, Germany) that was equipped with a spinning-disc device (YOKOGAWA CSU-X1 5000) and a cooled digital charge-coupled device (CCD) camera (AxioCam MRm). The GFP signal was excited using the 488-nm line of an Ar-Kr laser (Zeiss), and images collected via the Zen 2012 (Blue edition) software platform.

### Evaluation of cytotoxicity

To detect cytotoxic effects of the *E. lata* secondary metabolites and their analogues on Vrup GFP-TuB6 cells, we used the Evans Blue Dye Exclusion Assay [46]. Cell mortality was scored with an Axioskop microscope (Zeiss, Jena, Germany), equipped with a 32× objective (Zeiss Neofluar, Jena, Germany), and a digital CCD camera (AxioCam MRm). Mortality was determined as the percentage of blue cells among total counted cells. Each data point represents the average and standard error from at least 1000 cells counted and collected from at least three independent experimental series.

## Acknowledgements

This work was supported by the protect of the National Natural Science Foundation of China (32102308), Inteeref Upper Rhine Science Offensive (project DialogProtec) and by a fellowship from the China Scholarship Council to Pingyin Guan.

## Data availability

Data are available upon request to the corresponding author.

## Conflict of interest

The authors have no conflicts of interest to declare.

## Supplementary data

Supplementary data is available at *Horticulture Research* online.

## Supplementary Material

Web_Material_uhac120
