# Supplementary material for: The fungal elicitor eutypine from Eutypa lata activates basal immunity through its phenolic side chains
Source: Hortic Res. 2022 Jun 1;9:uhac120. doi: 10.1093/hr/uhac120 (PMC9343913; doi:10.1093/hr/uhac120)
Supplement: Web_Material_uhac120 [file web_material_uhac120.docx]

**The fungal elicitor eutypine from *Eutypa lata* activates basal immunity through its** **phenolic side chains**

Pingyin Guan^1,2^, Florian Schmidt^3^, Jochen Fischer^3^, Michael Riemann^2^, Eckhard Thines^3^ and Peter Nick^2,*^

^1^College of Horticulture, China Agricultural University, Beijing 100193, China

^2^Molecular Cell Biology, Botanical Institute, Karlsruhe Institute of Technology, Fritz-Haber-Weg 4, 76131 Karlsruhe, Germany

^3^Institut für Biotechnologie und Wirkstoff-Forschung gGmbH, Hanns-Dieter-Hüsch-Weg 17, 55128 Mainz, Germany

* Correspondence: Prof. Dr. Peter Nick, Molecular Cell Biology, Botanical Institute, Karlsruhe Institute of Technology, Fritz-Haber-Weg 4, 76131 Karlsruhe, Germany phone +49 721 608 42144, peter.nick@kit.edu

**Supplementary data:**

**Table S1.** List of oligonucleotide primers used for expression analysis in grapevine and tomato by quantitative PCR.

Primers used in grapevine experiments:

**
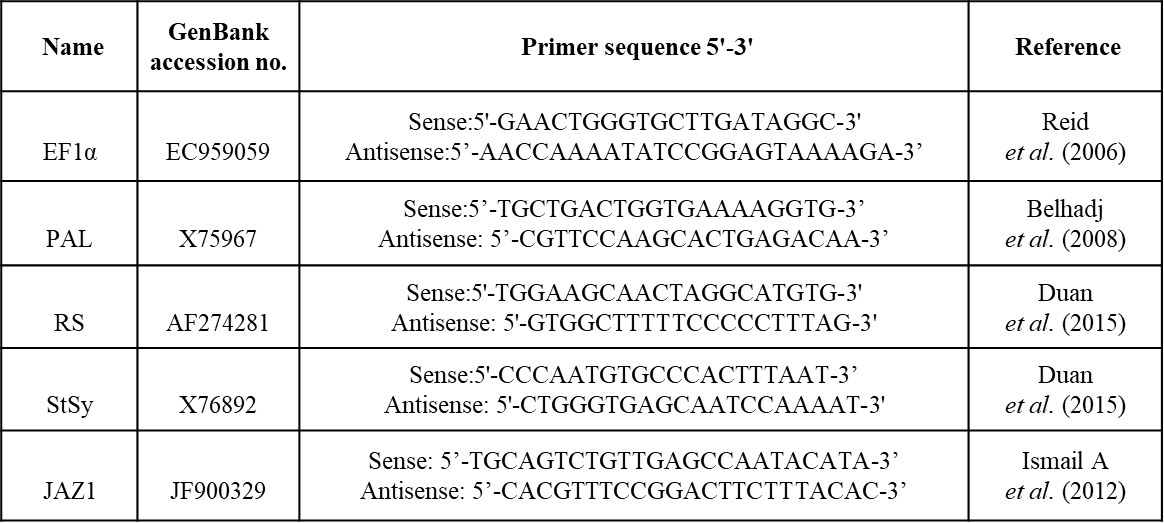
**

(EF1α, elongation factor 1α; PAL, phenylalanine ammonia lyase; RS, resveratrol synthase; StSy, stilbene synthase; JAZ1, the jasmonate ZIM/tify-domain protein 1, a transcriptional repressor)

Primers used in tomato experiments:

| **Name** | **NCBI Accession No.** | **Primer sequence (5’-3’)** |
| --- | --- | --- |
| Actin^*^ | AB199316.1 | Sense: 5’-CTCGAGCAGTGTTTCCCAGT-3’ |
|  |  | Antisense: 5’-GGTGCCTCAGTCAGGAGAAC-3’ |
| SOD | M37151 | Sense: 5’-TGTTGAGGGGGTTGTCACTC-3’ |
|  |  | Antisense: 5’-CGTGAAGTCCAGGAGCAAGT-3’ |
| PR1b1 | Y08804.1 | Sense: 5’-TGTCCGAGAGGCCAAGCTA-3’ |
|  |  | Antisense: 5’-TGTCCGATCCAGTTGCCTA-3’ |
| PR2b | NM_001247876.2 | Sense: 5’-ATCAGCCCTGTTACTGGCAC-3’ |
|  |  | Antisense: 5’-GGTGCACGTGTATCCCTCAA-3’ |
| CHI9 | NM_001247474.2 | Sense: 5’-TGGGGATATCACTGCCCGTA-3’ |
|  |  | Antisense: 5’-CCGTAGGCCATCCTCCAGTA-3’ |
| PR5 | NM_001247422.3 | Sense: 5’-CGGTGATTGTGGTGGAGTCT-3’ |
|  |  | Antisense: 5’-CCTCCGGGTACCCTAAGTGA-3’ |
| ICS | DQ984132.1 | Sense: 5’-GAAAAGGGTCTTGGCGCACT-3’ |
|  |  | Antisense: 5’-TAAATACGATGCGGCAGGC-3’ |
| NPR1 | KX198701.1 | Sense: 5’-TTGCACAAGTTGATGGCACG-3’ |
|  |  | Antisense: 5’-TCCACTGTTGTCCTCTGTGC-3’ |
| TGA1a | XM_004236682.3 | Sense: 5’-CTTGCTCAAAACCGTGAGGC-3’ |
|  |  | Antisense: 5’-CCTGTTTTCTGGCCCGATCT-3’ |
| TGA2 | U318626 | Sense: 5’-CCGATGGTTGGAAGAGCAG-3’ |
|  |  | Antisense: 5’-CCACATCCCCCTTTATC-3’ |


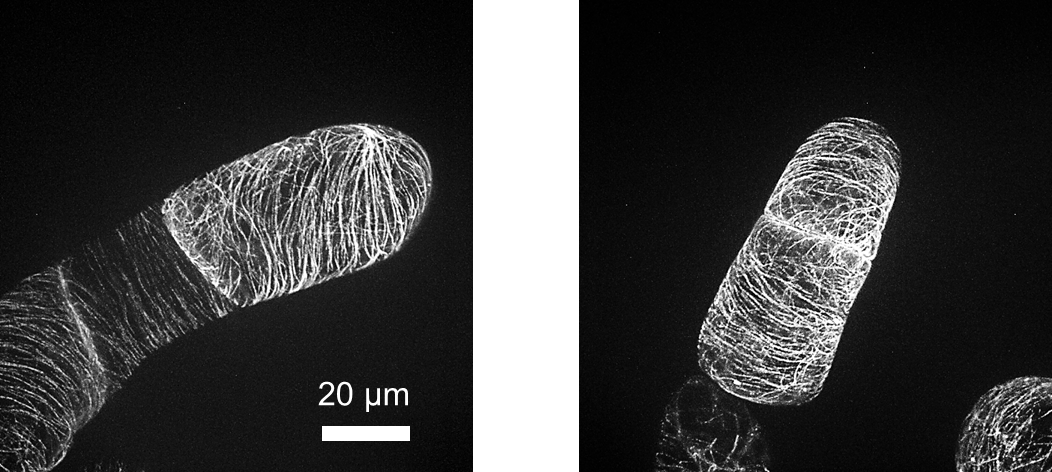


**Figure S1.** Microtubular responses to methanol control (left) and eutypinol (right) in grapevine model *V. rupestris* TuB6-GFP for 8 hours. Cells were challenged 10 µM eutypinol (right) for 8 hours. 0.1% methanol was chosen as the solvent control (left). Two treatments were all emerged in cortical microtubules, respectively. Observations were based on at least four independent experimental series with a population of 50 individual cells for each treatment. Bars, 20 μm.


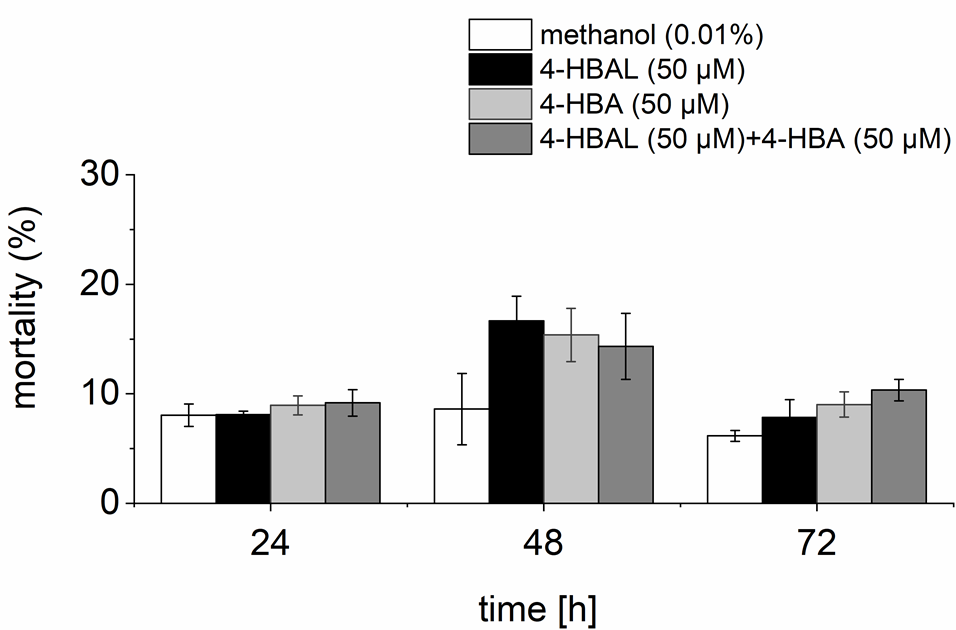


**Figure S2.** Response of mortality in the cellular grapevine model *V. rupestris* TuB6-GFP to chemical analogues of acetylenic phenols secreted by *Eutypa lata.* 4-HBAL, 4-HBA，and their combination were administered in a concentration of 50 µM for the indicated time intervals. Treatment with 0.01% methanol was used as solvent control. Mortality was scored using the Evans Blue dye exclusion assay. Data represent means ± SE from 1500 individual cells sampled in three independent experimental series. Significant differences are indicated by * (*P*< 0.05), ** (*P*< 0.01), or *** (*P*< 0.001) based on a homoscedastic Student’s t-test.


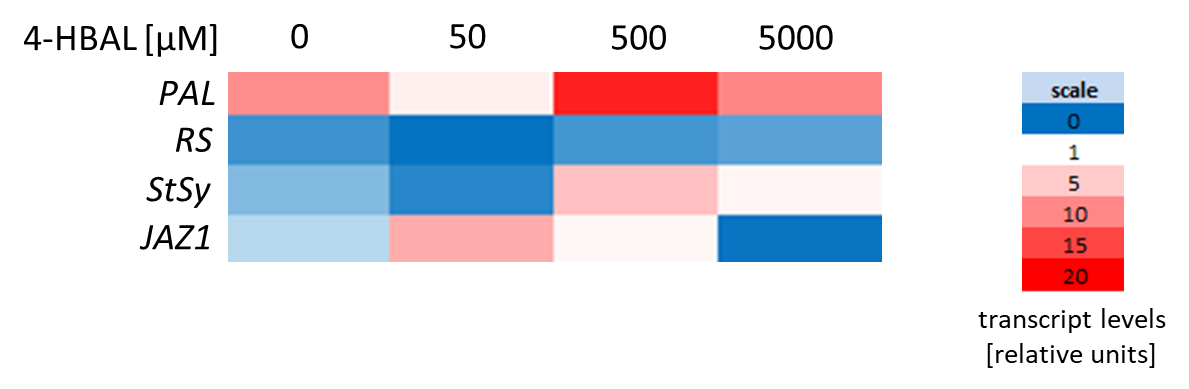


**Figure S3.** Response of defence genes to eutypine analogue 4-HBAL treatment in *Vitis vinifera* L.Tianyuanqi leaves. The grapevine leaves were sprayed with 50 µM, 500 µM or 5000 µM 4-HBAL for one hour. The expression defence genes (*PAL*, *RS*, *StSy, JAZ1)* was evaluated by qPCR. And gene *elongation factor 1α* (*EF1α*) was used as the endogenous reference gene. Data represent mean ± standard error (SE) from three independent experimental series, each in technical triplicates.


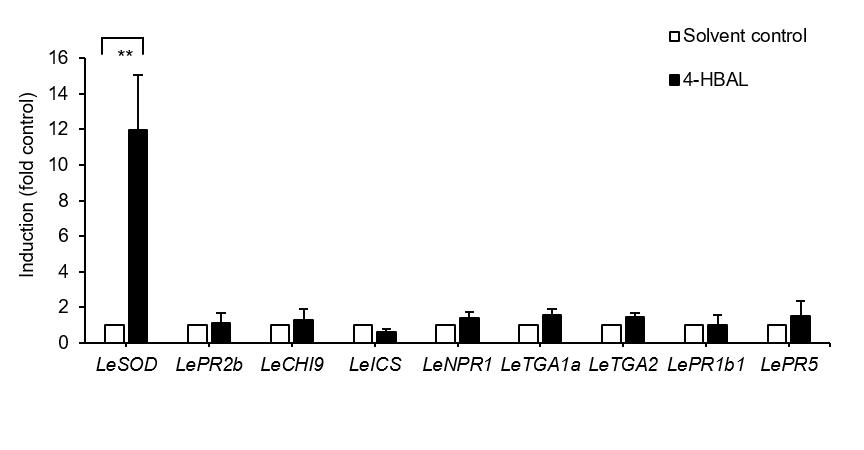


**Figure S4.** Quantitative RT-PCR analysis for the defence-related genes expression in response to eutypine analogue 4-HBAL treatment in ‘Micro-Tom’ tomato. Relative transcript accumulation of superoxide dismutase (SOD), β-1,3-glucanase (PR2b) and chitinase (CHI9), isochorismate synthase (ICS), the non-inducible pathogenesis-related 1 (NPR1), TGA transcription factors 1a (TGA1a), TGA transcription factors 2 (TGA2), pathogenesis-related protein gene 1b1 (PR1b1), and pathogenesis-related protein gene 5 (PR5). Values were normalized to solvent control after one hour 4-HBAL (500 µM) treatment, and actin was used as the endogenous reference gene. Vertical bars represent the standard errors of three replicates. Significant differences are indicated by ** (P< 0.01) based on a homoscedastic Student’s t-test.


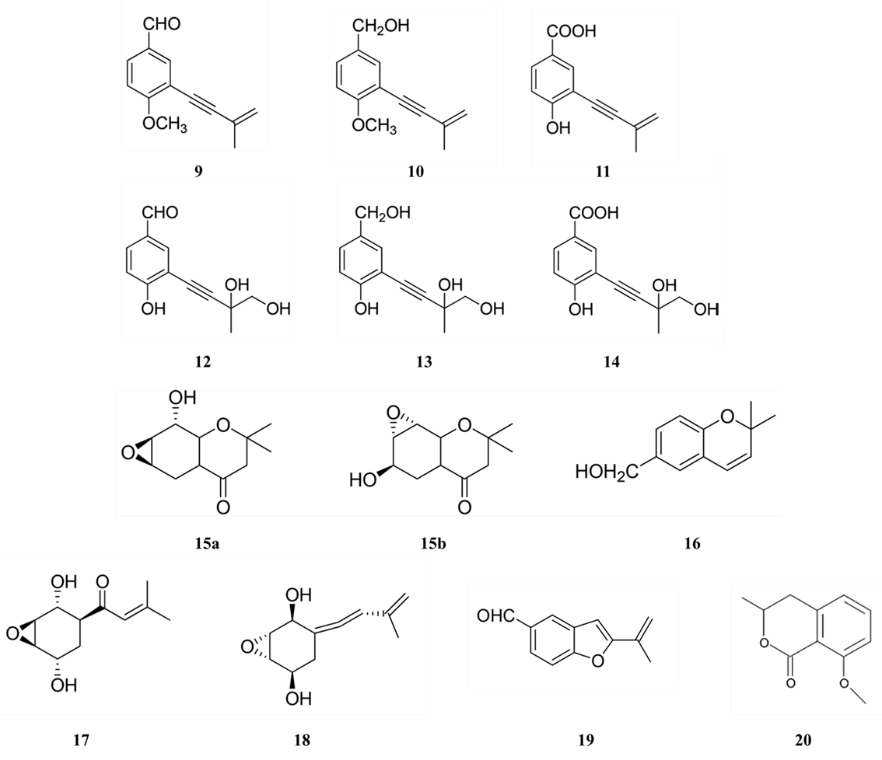


**Figure S5.** Structures of phenol (9-14), chromanone (15-16), cyclohexene oxide (17-18) derivatives, 2-isopropenyl-5-formylbenzofuran (19) and O-methylmellein (20) metabolites produced by *Eutypa lata* fungi involved in GTDs
